# Geriatric nutritional risk index as a predictor of 30-day and 365-day mortality in patients with acute myocardial infarction: a retrospective cohort study using the MIMIC-IV database

**DOI:** 10.3389/fnut.2025.1544382

**Published:** 2025-02-05

**Authors:** Xiaolong Zheng, Xin Zheng, Changgui Zhang, Minghua Liu

**Affiliations:** ^1^Emergency Department, The First Hospital Affiliated to Army Medical University, Chongqing, China; ^2^Department of Orthopedics, the 963rd Hospital of the Joint Service Support Force of the PLA, Jiamusi, China; ^3^Emergency Department, the 963rd Hospital of the Joint Service Support Force of the PLA, Jiamusi, China

**Keywords:** geriatric nutritional risk index, acute myocardial infarction, mortality, landmark analysis, mediation analysis, cox regression model

## Abstract

**Background:**

The Geriatric Nutritional Risk Index (GNRI) is a clinical indicator for evaluating the nutritional status of patients, but its role in the short-term prognosis of patients with acute myocardial infarction is still not fully understood. This study aims to explore the correlation between the GNRI and the overall mortality within 30 days and 365 days in those with acute myocardial infarction (AMI).

**Methods:**

A retrospective analysis was performed utilizing the Medical Information Mart for Intensive Care IV (MIMIC-IV) database. The study included 895 patients diagnosed with AMI and identified through ICD9 and ICD10 codes (410, I21, I23) who were hospitalized for the first time due to AMI. Subjects were classified into four groups according to GNRI: high (GNRI <82, *n* = 110), moderate (82 ≤ GNRI <92, *n* = 205), low (92 ≤ GNRI <98, *n* = 225), and no nutritional risk (GNRI ≥98, *n* = 355). Restricted cubic splines (RCS) and threshold effect analyses were applied to explore the non-linear relationship between GNRI and mortality. Subgroup analyses were conducted based on gender, hypertension, diabetes, stroke, hyperlipidemia, chronic obstructive pulmonary disease, and age. A mediation study was conducted to investigate the impact of lymphocytes on the association between GNRI and mortality.

**Results:**

In an overall sample of 895 patients, an elevated GNRI correlated with reduced 30-day (HR = 0.937, 95% CI: 0.917–0.957, *p* < 0.001) and 365-day mortality (HR = 0.937, 95% CI: 0.923–0.950, *p* < 0.001). The trend analysis for GNRI categories indicated a significant decline in mortality associated with rising GNRI (P for trend <0.001). Subgroup analysis validated the consistency of such results throughout diverse patient characteristics. The lymphocytes significantly mediated the relationship between GNRI and 30-day mortality (ACME: 0.022, 95% CI: 0.003–0.180, *p* < 0.001). A landmark analysis at 20 days after admission further demonstrated the impact of GNRI on mortality during different phases of recovery.

**Conclusion:**

This study highlights the prognostic value of GNRI in predicting short-term and long-term mortality in AMI patients, emphasizing the significance of nutritional status and inflammatory indicators in the therapy and risk assessment of these individuals.

## 1 Introduction

Acute myocardial infarction (AMI) is primarily caused by the formation of thrombi within the coronary arteries, leading to irreversible necrosis of myocardial cells (1, 2). The pathophysiological changes it induces mainly include acute myocardial cell injury, inflammatory response, microcirculatory disorders, ventricular remodeling, and electrophysiological instability during the acute phase (3). Patients experiencing cardiogenic shock due to myocardial infarction have a 30-day mortality rate of roughly 40%, with a one-year mortality rate nearing 50% (4). In the early stages of AMI, platelet aggregation and thrombus formation occur as a result of plaque rupture or erosion within the coronary artery, subsequently causing acute coronary occlusion (5). As the ischemic time extends, myocardial cells begin to suffer irreversible damage, ultimately leading to apoptosis or necrosis (6, 7).

The systemic nutritional status is pivotal in the occurrence and prognosis of AMI. A study revealed that of 10,161 AMI patients that received percutaneous coronary intervention (PCI), over 50% displayed malnutrition (8). Good nutritional status can provide adequate energy support, promoting the repair and regeneration of damaged myocardial tissue (9, 10). The Geriatric Nutritional Risk Index (GNRI) is initially created to evaluate the nutritional condition of elderly people (11). Research indicates that the GNRI not only accurately assesses the nutritional status of older individuals but also corresponds with the prognosis of several chronic diseases, including chronic kidney disease, heart failure, and pulmonary disorders (12). Prior investigations have suggested that patients with diminished nutritional scores face heightened risks of adverse cardiovascular events and overall mortality (9, 13, 14). The study on elderly AMI patients found that the all-cause mortality rate in the low GNRI group was significantly higher than that in the high GNRI group, and GNRI was an independent predictor of long-term mortality (13). However, those studies have certain methodological limitations; most did not employ mediation analysis to conduct in-depth exploration on the complex relationship between nutritional status and AMI prognosis, nor did they use landmark analysis to evaluate the dynamic impact of nutritional status changes at different follow-up time points on prognosis. Additionally, there were limitations such as insufficient sample sizes, lacking the ability to identify potential mechanisms by which nutritional status affects prognosis and to assess differences in its effects at different time points. Based on the above background, this study will further investigate the mediating effect of inflammatory cells between GNRI and the short-term prognosis of AMI, aiming to provide more comprehensive guidance for clinical practice, aiming to promote the application of nutritional assessment in the management of cardiovascular diseases and provide novel insights for enhancing clinical outcomes in AMI patients. We hypothesized that higher GNRI values are associated with lower short-term (30 days) and long-term (365 days) mortality risks; moreover, we speculate that lymphocyte count may mediate the relationship between GNRI and mortality.

## 2 Materials and methods

### 2.1 Study population

**Figure 1:**
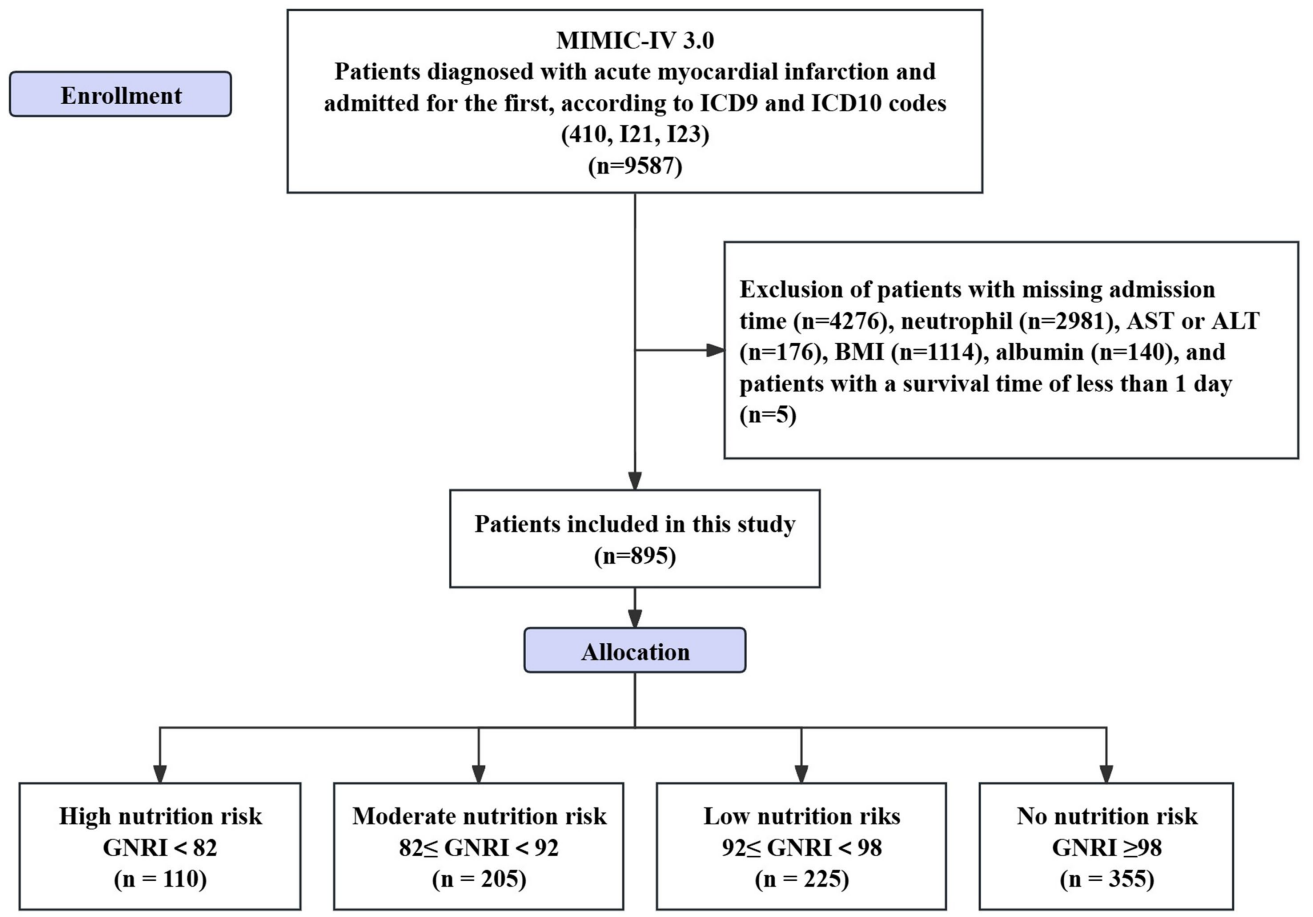
Flowchart of patient inclusion and exclusion.

This is a retrospective study. The data applied in the research is from the Medical Information Mart for Intensive Care IV (MIMIC-IV 3.0) database, developed by the Laboratory of Computational Physiology in Massachusetts.^1^ Inclusion criteria: (1) patients diagnosed with AMI as identified by International Classification of Diseases, Ninth Revision (ICD-9) (410) and ICD-10 codes (I21, I23); (2) individuals committed to hospitals for the initial occurrence of AMI. Exclusion criteria: (1) patients lacking information on admission time or with a survival time of less than 1 day; (2) patients without protein test results; (3) patients without Body Mass Index (BMI) data; (4) patients lacking complete blood count or biochemical test results (see Figure 1). One of the authors of this study, Zheng, successfully completed the collaborative training program course titled “Research with Data or Specimens” under the Collaborative Institutional Training Initiative (CITI) project, passed the examination (Record ID: 39635575), and subsequently obtained permission to access the dataset.

Given that the MIMIC-IV database is publicly available and contains de-identified patient information, neither informed permission nor ethical approval were needed for this investigation.

### 2.2 Data extraction

The extraction of data was conducted via Decisionlinnc1.071 software.^2^ The following patient demographic data were collected: gender, age, and BMI. Additionally, information on past medical history, including hypertension, stroke, hyperlipidemia, chronic obstructive pulmonary disease (COPD), and diabetes were gathered. All relevant diseases were identified using the ICD-9, in conjunction with the ICD-10 diagnostic codes, derived from the patient discharge records in the MIMIC-IV database. Laboratory test results were also collected, including absolute lymphocyte count, arterial carbon dioxide partial pressure (PaCO_2_), arterial oxygen partial pressure (PaO_2_), hematocrit, blood glucose, creatinine, chloride, alanine aminotransferase (ALT), red blood cell count, total bilirubin, blood urea nitrogen (BUN), absolute neutrophil count, aspartate aminotransferase (AST), hemoglobin, total calcium, red cell distribution width (RDW), triglycerides, albumin, white blood cell count, and lactate. Survival status at 30 days and 365 days after admission was also obtained. All laboratory parameters were extracted from the first biochemical analysis performed after admission. For continuous variables, outliers were detected using box plots, and these outliers were subsequently removed from the dataset for further analysis.

### 2.3 Defining of nutritional status and outcomes

This study adopted a simplified method for calculating the GNRI as reported before. The GNRI was determined using the formula GNRI = [14.89 × serum albumin (g/dL)] + [41.7 × actual BMI/ideal BMI], where the ideal BMI was defined as 22 kg/m2. In cases where a patient’s BMI surpassed the ideal value, the ratio of “actual BMI/ideal BMI” was standardized to 1 (14, 15). The principal endpoint of the study was the 30-day death rate post-admission, while the secondary outcome was the 365-day mortality rate. The 30-day mortality rate is a widely accepted standard in cardiology for evaluating the immediate outcomes following an acute event such as AMI. It provides insights into the effectiveness of initial treatments and interventions during the critical early phase of recovery. On the other hand, 365-day mortality offers a broader perspective on long-term survival, capturing the impact of both short-term and sustained factors that influence patient prognosis over a full year.

### 2.4 Statistical analysis

The data for continuously distributed normally distributed variables were shown as the mean ± standard deviation (x̅ ± s). To evaluate the normality of continuous data, the Shapiro–Wilk test was utilized. Student’s t-test was carried out for group comparisons. Regarding categorical variables, descriptive statistics were presented in the form of percentages and frequencies. The chi-square test was employed to make group comparisons. Three distinct cox regression models were developed. The initial Model 1 was a fundamental model, which did not account for any covariates. Model 2 was formulated based on Model 1, incorporating age and sex as covariates for adjustment, so as to rule out the possible confounding impacts of these two elements on the outcomes. Model 3 was further expanded on this by incorporating additional variables: hypertension, hyperlipidemia, COPD, lymphocyte count, RDW, creatinine, urea nitrogen, PaO_2_, sodium, beta-blocker, antiplatelet and calcium, to more comprehensively assess the influence of GNRI under the combined effect of multiple factors. The GNRI was divided into four levels from low to high as a categorical variable referring to previous studies (15). Based on this, a Kaplan–Meier survival analysis method was developed to clarify the disparities in all-cause mortality among groups, utilizing the log-rank test to assess inter-group differences. Additionally, the trend effect of different levels of GNRI in the Cox regression models was assessed. A four-knot restricted cubic spline (RCS) curves and threshold effect analysis were used to study the non-linear correlation. Additionally, subgroup analyses were performed. Mediation analysis was performed using the “mediation” package in R language to evaluate the mediating role of inflammatory markers (lymphocytes). Moreover, landmark analysis was used to separately examine the associations of GNRI with mortality within 20 days and after 20 days (20–30 days), as well as within 240 days and after 240 days (240–365 days). Finally, subgroup analyses were conducted based on gender, hypertension, diabetes, stroke, hyperlipidemia, COPD, and age.

The present study’s statistical analysis was carried out with the utilization of R Studio (R4.1.0 version) and Decision Chain (4.1 version). A two-tailed *p* value below 0.05 was regarded as statistically significant.

## 3 Results

### 3.1 Patient characteristics

This study encompassed 895 patients who had AMI. Significant disparities in demographic features were manifested among the groups, specifically in relation to age, BMI, the distribution of genders, as well as the medical histories of hypertension, diabetes mellitus, stroke, hyperlipidemia, and COPD (*p* < 0.05; Table 1).

**Table 1:** Patients’ general clinical characteristics.

Characteristics	High nutritional risk GNRI<82	Moderate nutritional risk 82 ≤ GNRI <92	Low nutritional risk 92 ≤ GNRI <98	No nutritional risk GNRI≥98	*p* value
Number, n	110	205	225	355	
Age (year)	68.68 ± 13.79	72.03 ± 13.24	70.35 ± 12.07	65.91 ± 11.17	<0.001
BMI (kg/m^2^)	26.68 ± 6.37	26.56 ± 5.61	28.78 ± 5.32	29.20 ± 5.23	<0.001
Gender (n, %)					<0.001
Female	54 (49.1%)	86 (42.0%)	68 (30.2%)	78 (22.0%)	
Male	56 (50.9%)	119 (58.0%)	157 (69.8%)	277 (78.0%)	
Hypertension (n, %)					<0.001
No	83 (75.5%)	149 (72.7%)	141 (62.7%)	166 (46.8%)	
Yes	27 (24.5%)	56 (27.3%)	84 (37.3%)	189 (53.2%)	
Diabetes (n, %)					0.751
No	64 (58.2%)	125 (61.0%)	126 (56.0%)	203 (57.2%)	
Yes	46 (41.8%)	80 (39.0%)	99 (44.0%)	152 (42.8%)	
Stroke (n, %)					0.574
No	103 (93.6%)	190 (92.7%)	209 (92.9%)	338 (95.2%)	
Yes	7 (6.36%)	15 (7.32%)	16 (7.11%)	17 (4.79%)	
Hyperlipidemia (n, %)					<0.001
No	62 (56.4%)	92 (44.9%)	86 (38.2%)	115 (32.4%)	
Yes	48 (43.6%)	113 (55.1%)	139 (61.8%)	240 (67.6%)	
COPD (n, %)					0.001
No	85 (77.3%)	166 (81.0%)	190 (84.4%)	322 (90.7%)	
Yes	25 (22.7%)	39 (19.0%)	35 (15.6%)	33 (9.30%)	
Red blood cell (10^12^/L)	3.47 ± 0.81	3.68 ± 0.70	3.99 ± 0.72	4.39 ± 0.64	<0.001
Lymphocyte count (10^9^/L)	1.16 ± 0.69	1.51 ± 0.86	1.69 ± 0.88	1.94 ± 0.85	<0.001
Neutrophil count (10^9^/L)	10.04 ± 5.07	8.80 ± 4.50	9.21 ± 4.09	9.02 ± 3.82	0.083
RDW (%)	14.39 ± 1.28	14.19 ± 1.37	13.88 ± 1.30	13.46 ± 1.17	<0.001
Hemoglobin (g/dL)	10.29 ± 2.33	10.92 ± 2.08	11.77 ± 2.07	13.11 ± 1.90	<0.001
Platelet (10^9^/L)	182.32 ± 71.02	190.65 ± 62.24	201.39 ± 56.77	207.26 ± 53.87	<0.001
ALT (IU/L)	26.87 ± 16.68	25.81 ± 15.19	24.57 ± 13.21	27.65 ± 14.42	0.087
AST (IU/L)	35.83 ± 23.66	37.03 ± 24.84	35.89 ± 24.85	34.23 ± 20.47	0.559
Creatinine (mg/dL)	1.15 ± 0.41	1.10 ± 0.38	1.10 ± 0.36	1.00 ± 0.27	<0.001
Urea nitrogen (mg/dL)	25.62 ± 12.35	24.47 ± 11.91	23.01 ± 10.77	19.25 ± 8.82	<0.001
Total bilirubin (mg/dL)	0.60 ± 0.32	0.61 ± 0.33	0.59 ± 0.29	0.61 ± 0.31	0.845
Albumin (g/dL)	2.39 ± 0.35	3.14 ± 0.23	3.57 ± 0.11	4.08 ± 0.24	<0.001
Lactate (mmol/L)	1.50 ± 0.46	1.52 ± 0.55	1.46 ± 0.51	1.51 ± 0.50	0.651
PaO_2_ (mmHg)	123.71 ± 115.22	177.67 ± 138.44	238.02 ± 144.84	309.56 ± 128.18	<0.001
PaCO_2_ (mmHg)	39.13 ± 6.88	39.42 ± 6.45	40.59 ± 5.64	40.32 ± 5.43	0.055
Potassium (mmol/L)	4.19 ± 0.56	4.13 ± 0.48	4.20 ± 0.45	4.17 ± 0.40	0.448
Sodium (mmol/L)	137.90 ± 4.12	138.20 ± 3.78	138.62 ± 3.28	139.11 ± 3.01	0.002
Chloride(mmol/L)	102.10 ± 5.23	101.81 ± 4.59	101.68 ± 3.97	102.08 ± 3.29	0.646
Calcium total (mmol/L)	8.08 ± 0.70	8.49 ± 0.59	8.71 ± 0.59	8.98 ± 0.60	<0.001
ACEI/ARB use (n, %)	15 (13.64%)	48 (23.41%)	50 (22.22%)	76 (21.41%)	0.210
Diuretic use (n, %)	10 (9.09%)	24 (11.71%)	34 (15.11%)	35 (9.86%)	0.216
Beta-blocker use (n, %)	88 (80.00%)	172 (83.90%)	202 (89.78%)	333 (93.80%)	<0.001
Antiplatelet use (n, %)	87 (79.09%)	172 (83.90%)	211 (93.78%)	341 (96.06%)	<0.001
Statin use (n, %)	11 (10.00%)	20 (9.76%)	24 (10.67%)	27 (7.61%)	0.609
GNRI	76.06 ± 5.03	87.60 ± 2.65	94.77 ± 1.64	102.34 ± 3.57	<0.001
30-day mortality (n, %)					<0.001
No	89 (80.9%)	184 (89.8%)	207 (92.0%)	344 (96.9%)	
Yes	21 (19.1%)	21 (10.2%)	18 (8.00%)	11 (3.10%)	
365-day mortality (n, %)					<0.001
No	70 (63.6%)	148 (72.2%)	185 (82.2%)	333 (93.8%)	
Yes	40 (36.4%)	57 (27.8%)	40 (17.8%)	22 (6.20%)	

BMI, body mass index; COPD, chronic obstructive pulmonary disease; RDW, red cell distribution width; ALT, alanine aminotransferase; AST, aspartate aminotransferase; GNRI, geriatric nutritional risk index.

### 3.2 Survival analysis

**Figure 2:**
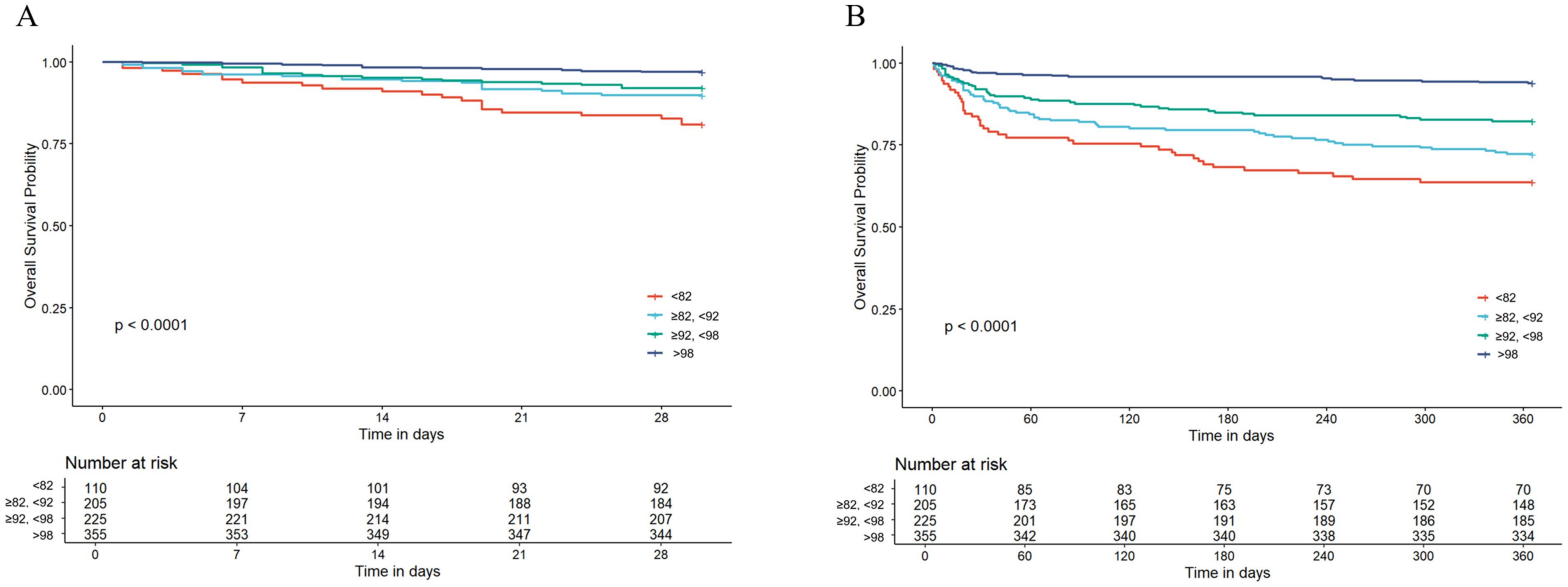
The Kaplan–Meier survival analysis curve for all-cause mortality. **(A)** Intergroup comparison of mortality rates within 30 days, **(B)** intergroup comparison of mortality rates within 365 days.

The survival curves for AMI patients up to 30 days after hospitalization showed a clear diverging trend, and the log-rank test further confirmed that these inter-group differences were statistically significant (*p* < 0.0001; Figure 2A). Similar to the 30-day results, the survival curves of the groups also exhibited significant differences at later time points. The disparities detected among the groups were of a notably high statistical significance (*p* < 0.0001; Figure 2B).

### 3.3 The detection of nonlinear relationships

**Figure 3:**
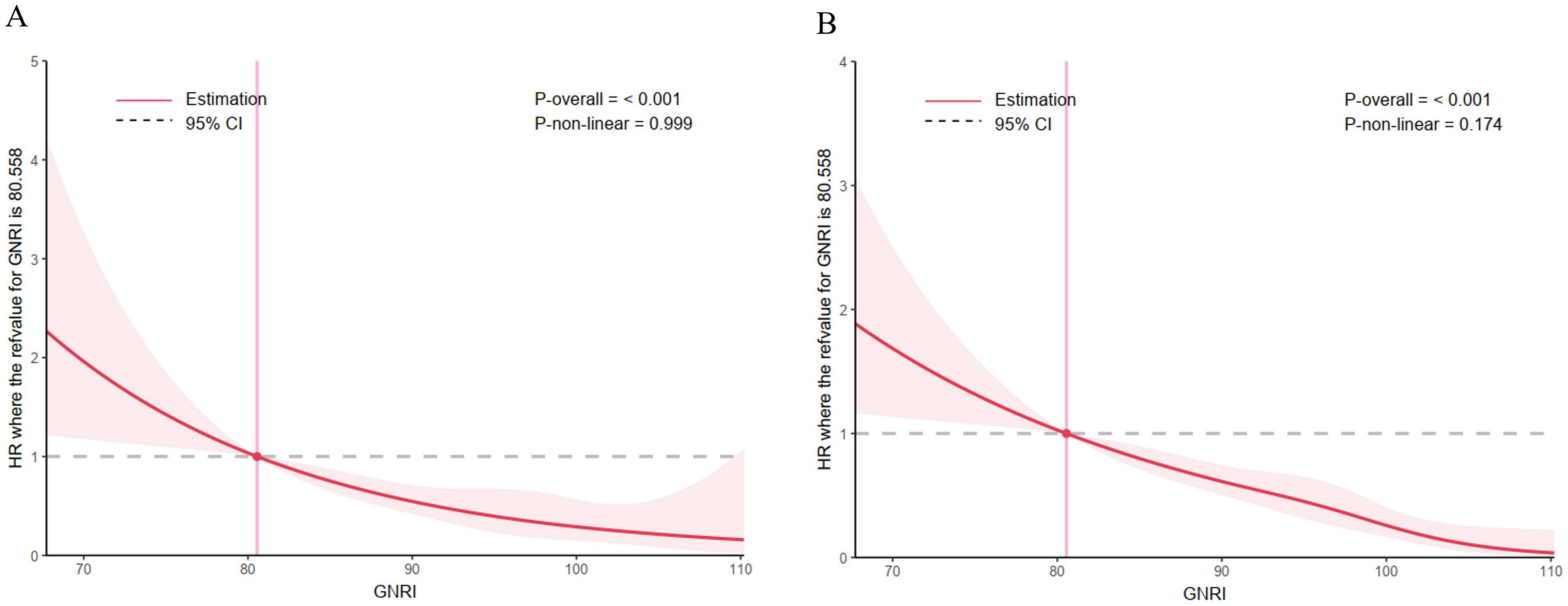
Correlation between the GNRI and the hazard ratio for all-cause mortality in patients with acute myocardial infarction. **(A)** The restricted cubic spline curve for the 30-day mortality rate, **(B)** the restricted cubic spline curve for the 365-day mortality rate.

The RCS curves failed to show any sign of a non-linear relationship within 30 days or 365 days among AMI patients after hospitalization (all *p* > 0.05). As the GNRI value increases, mortality showed a decreasing trend (Figure 3).

### 3.4 Cox regression of GNRI and 30-day and 365 day mortality

In Model 1, which served as the baseline model without any covariate adjustment, a substantial correlation was noted between the GNRI index and mortality at both 30 days and 365 days. With every single-unit increment in the GNRI index, the hazard ratio (HR) pertaining to 30-day mortality declined by 6.3% (HR: 0.937, 95% confidence interval [CI]: 0.917–0.957, *p* < 0.001), and in the case of 365-day mortality, it lessened by 6.3% (HR: 0.937, 95% CI: 0.923–0.950, *p* < 0.001). When stratifying patients by GNRI categories, those with lower GNRI scores exhibited progressively higher risks of mortality. The *p*-value for trend indicated a statistically significant decrease in mortality risk with increasing GNRI scores in relation to both the 30-day and 365-day mortality (both *p* < 0.001). In Model 2, the significant associations between the GNRI index and both mortality endpoints persisted. The *p*-value for trend remained statistically significant for both time points (*p* < 0.001). In Model 3, which further adjusted for a range of potential confounders, the GNRI index continued to demonstrate a significant association with 365-day mortality (HR: 0.965, 95% CI: 0.946–0.985, *p* < 0.001). Nevertheless, in the case of 30-day mortality, although the GNRI index was still in a protective direction (HR: 0.972, 95% CI: 0.944–1.001), the correlation was not statistically significant any longer (*p* = 0.060). The stratified analysis by GNRI categories showed similar trends, with statistically significant reductions in mortality risk for most GNRI categories at 365 days but with less consistent results at 30 days. The *p*-value for the trend analysis indicated statistical significance for 365-day mortality (*p* = 0.002), whereas it did not reach statistical significance for 30-day mortality (*p* = 0.266; Table 2).

**Table 2:** Cox regression of GNRI and 30-day and 365-day mortality in patients.

	Model 1	*p* value	Model 2	*p* value	Model 3	*p* value
30-day mortality						
GNRI index	0.937 (0.917 ~ 0.957)	<0.001	0.938 (0.917 ~ 0.960)	<0.001	0.972 (0.944 ~ 1.001)	0.060
GNRI<82	Ref		Ref		Ref	
82 ≤ GNRI <92	0.516 (0.282 ~ 0.946)	0.032	0.410 (0.223 ~ 0.754)	0.004	0.482 (0.246 ~ 0.942)	0.033
92 ≤ GNRI <98	0.397 (0.212 ~ 0.745)	0.004	0.359 (0.191 ~ 0.676)	0.002	0.591 (0.286 ~ 1.219)	0.154
GNRI≥98	0.150 (0.072 ~ 0.310)	<0.001	0.176 (0.084 ~ 0.371)	<0.001	0.551 (0.228 ~ 1.331)	0.185
*P* for trend		<0.001		<0.001		0.266
365-day mortality						
GNRI index	0.937 (0.923 ~ 0.950)	<0.001	0.938 (0.923 ~ 0.953)	<0.001	0.965 (0.946 ~ 0.985)	<0.001
GNRI<82	Ref		Ref		Ref	
82 ≤ GNRI <92	0.707 (0.472 ~ 1.059)	0.093	0.570 (0.379 ~ 0.857)	0.007	0.665 (0.426 ~ 1.036)	0.071
92 ≤ GNRI <98	0.431 (0.278 ~ 0.669)	<0.001	0.398 (0.256 ~ 0.619)	<0.001	0.621 (0.380 ~ 1.016)	0.058
GNRI≥98	0.140 (0.083 ~ 0.236)	<0.001	0.166 (0.097 ~ 0.281)	<0.001	0.375 (0.202 ~ 0.698)	0.002
*P* for trend		<0.001		<0.001		0.002

Model 1: no covariates were adjusted. Model 2: age and gender were adjusted. Model 3: age, gender, hypertension, hyperlipidemia, COPD, lymphocyte, rdw, creatinine, urea nitrogen, PO_2_, sodium and calcium, beta-blocker and antiplatelet were adjusted. COPD, chronic obstructive pulmonary disease; GNRI, geriatric nutritional risk index.

### 3.5 Mediation analysis

**Figure 4:**
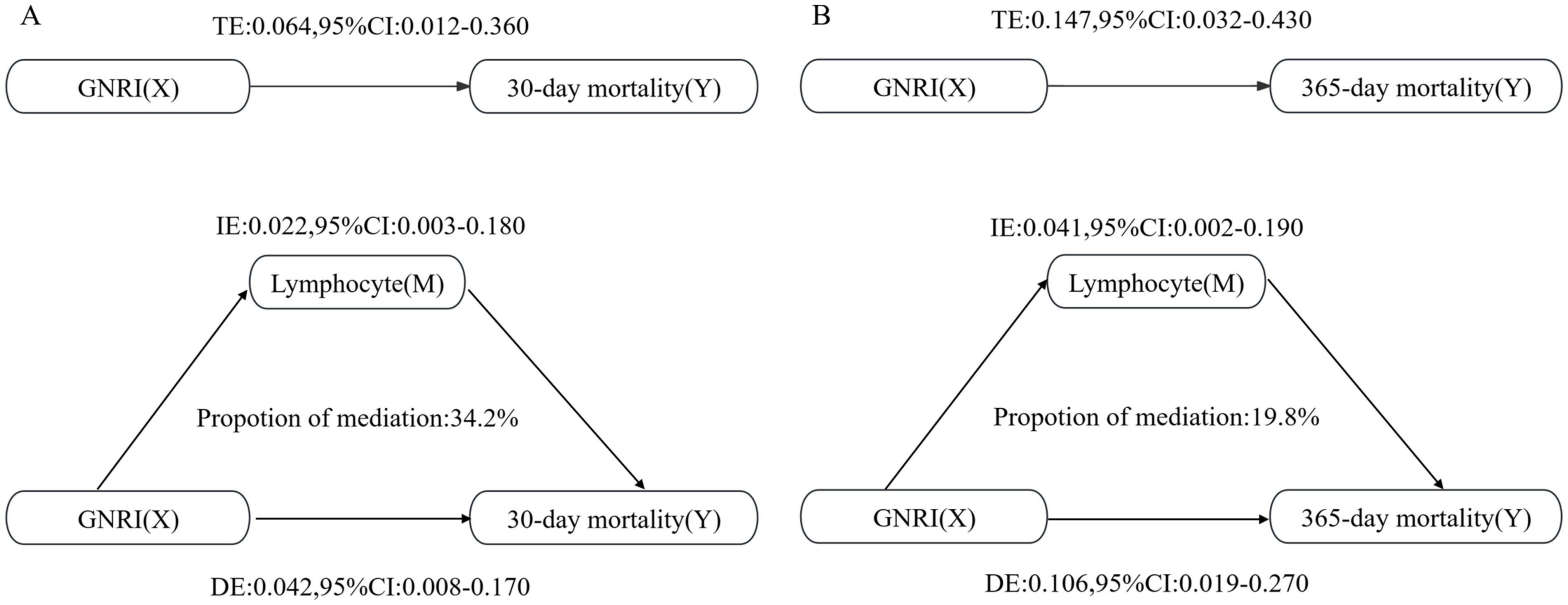
Mediation analysis of the association between GNRI and mortality rates, with lymphocytes as the mediator. **(A)** For the 30-day mortality analysis; **(B)** For the 365-day mortality analysis.

The fraction of the overall effect that was mediated by lymphocytes amounted to 0.342 (95% CI: 0.187–0.590), indicating that lymphocytes accounted for a substantial portion of the association between GNRI and 30-day mortality (Table 3). For 365-day mortality, similar patterns were observed. The ACME was also significant (Estimate: 0.002, 95% CI: 0.0001–0.020, *p* < 0.001). The ratio of the total effect which was mediated by lymphocytes was 0.230 (95% CI: 0.122–0.370; Figure 4).

**Table 3:** Lymphocytes as a mediator variable between GNRI and the correlation of all-cause mortality.

Mediation effect	Estimate	95% CI Lower	95% CI Upper	*p* value
30-day mortality				
Total effect	0.064	0.012	0.360	<0.001
ACME	0.022	0.003	0.180	<0.001
ADE	0.042	0.008	0.170	<0.001
Proportion mediated	0.342	0.187	0.590	<0.001
365-day mortality				
Total effect	0.008	0.001	0.080	<0.001
ACME	0.002	0.0001	0.020	<0.001
ADE	0.006	0.001	0.060	<0.001
Proportion mediated	0.230	0.122	0.370	<0.001

ACME, average causal mediation effect, ADE, average direct effect. GNRI, geriatric nutritional risk index.

### 3.6 Landmark analysis

For the period from admission to 20 days after hospitalization, GNRI was found to be significantly associated with mortality, with a HR of 0.937 (95% CI: 0.917–0.957, *p* < 0.001). This suggested that an elevated GNRI correlated with a diminished risk of mortality over the initial 20 days. Furthermore, in the comparison of patients having high GNRI levels with those possessing low GNRI levels, the HR was 0.267 (95% CI: 0.140–0.507, *p* < 0.001), further underscoring the protective effect of good nutritional status on early mortality. For the subsequent period from 20 to 30 days after admission, the association between GNRI and mortality remained significant, with an HR of 0.929 (95% CI: 0.889–0.971, *p* = 0.001). However, the magnitude of this association was slightly attenuated compared with the first 20 days (Table 4).

**Table 4:** Landmark analysis at 20 days of the association between GNRI and 30-day mortality.

Variable	0–20 days mortality		20–30 days mortality	
	HR (95%CI)	*p* value	HR (95%CI)	*p* value
GNRI	0.937 (0.917–0.957)	<0.001	0.929 (0.889–0.971)	0.001
High level vs. Low level	0.267 (0.140–0.507)	<0.001	0.325 (0.093–1.141)	0.079

GNRI, Geriatric Nutritional Risk Index; CI, confidence interval; HR, hazard ratio.

For the period from admission to 240 days after hospitalization, GNRI was found to be significantly associated with mortality, with a HR of 0.937 (95% CI: 0.923–0.950, *p* < 0.001). For the subsequent period from 240 to 365 days after admission, the association between GNRI and mortality remained significant, though with a slightly attenuated HR of 0.951 (95% CI: 0.917–0.987, *p* = 0.019). However, when comparing high versus low GNRI levels during this later time window, the HR was 0.383 (95% CI: 0.140–1.044, *p* = 0.065), suggesting a trend toward lower mortality among patients with better nutritional status that did not quite reach statistical significance (Table 5).

**Table 5:** Landmark analysis at 240 days of the association between GNRI and 365-day mortality.

Variable	0–240 days mortality		240–365 days mortality	
	HR (95%CI)	*p* value	HR (95%CI)	*p* value
GNRI	0.937 (0.923–0.950)	<0.001	0.951 (0.917–0.987)	0.019
High level vs. Low level	0.219 (0.139–0.343)	<0.001	0.383 (0.140–1.044)	0.065

GNRI, Geriatric Nutritional Risk Index; CI, confidence interval; HR, hazard ratio.

### 3.7 Subgroup analysis

**Figure 5:**
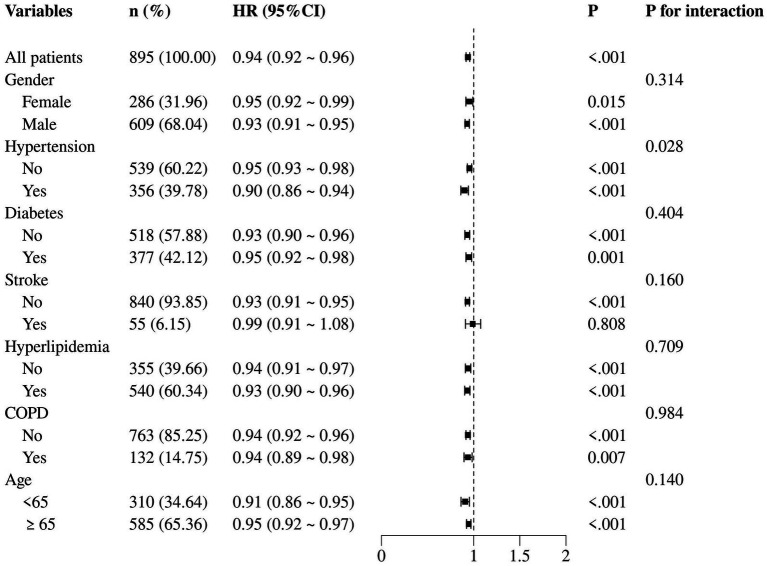
Subgroup analysis of the correlation between GNRI and 30-day all-cause mortality.

When examining the interaction between GNRI and each subgroup variable, no significant heterogeneity was observed across subgroups, as evidenced by the *p*-values for interaction which were all non-significant (*p* > 0.05). In the subgroup of female patients, the HR for the association between GNRI and 30-day mortality was 0.95 (95% CI: 0.92–0.99, *p* = 0.015), suggesting a similar but slightly attenuated protective effect of GNRI compared to the overall cohort. In contrast, among patients with hypertension, the HR was 0.90 (95% CI: 0.86–0.94, *p* < 0.001). Similar trends were observed for patients with and without diabetes, stroke, hyperlipidemia, and COPD. Notably, age did not significantly modify the association, as evidenced by the non-significant *p*-value for interaction (*p* = 0.140). This suggested that the prognostic value of GNRI was consistent across different age groups in AMI patients (Figure 5).

**Figure 6:**
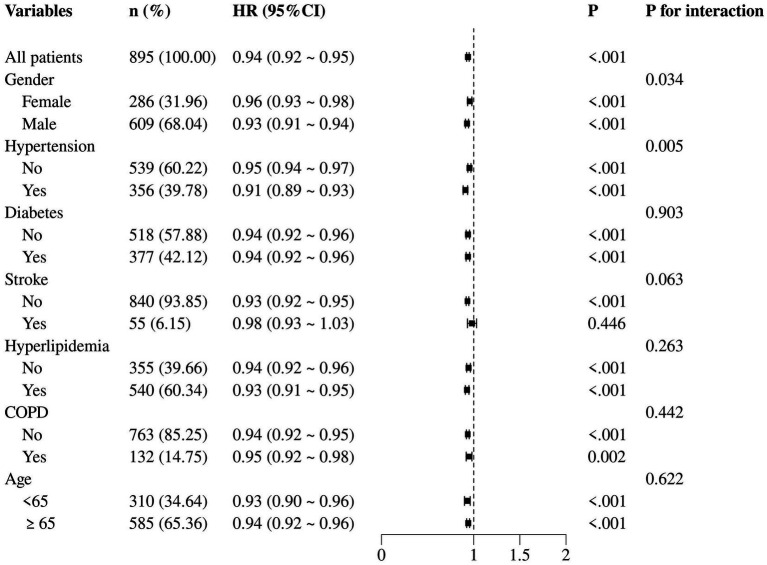
Subgroup analysis of the correlation between GNRI and 365-day all-cause mortality.

Extending the analysis to 365-day all-cause mortality, we observed a consistent pattern with the 30-day mortality results. Notably, among female patients, the HR for the association between GNRI and 365-day mortality was 0.96 (95% CI: 0.93–0.98, *p* < 0.001), indicating a slightly weaker but still significant protective effect of GNRI compared to the overall cohort. Conversely, in patients with hypertension, the HR was 0.91 (95% CI: 0.89–0.93, *p* < 0.001; Figure 6).

## 4 Discussion

This research investigated the association between GNRI and the mortality within 30 days and 365 days among persons with AMI. The results demonstrated that a higher GNRI was highly correlated with a decrease in both short-term and long-term mortality from all causes. This finding not only confirms the critical role of nutritional status in the prognosis of AMI patients but also suggests that GNRI, as a simple and practical tool for nutritional assessment, has the potential to become a new prognostic biomarker for AMI patients. Our study further elucidates the complex relationship between GNRI and AMI prognosis through the introduction of mediation analysis and landmark analysis, providing new perspectives and evidence for clinical practice.

In the management of AMI patients, nutritional status, as an important reflection of overall health, plays a significant role in patient outcomes (16). First, energy metabolism imbalance is one of the key mechanisms by which nutritional status affects AMI prognosis. After an AMI event, cardiomyocyte necrosis occurs due to ischemia and hypoxia, a process that consumes a large amount of energy (17). A good nutritional status can provide adequate energy substrates to support the repair and regeneration of cardiomyocytes (18). Patients with malnutrition frequently encounter imbalances in energy metabolism. Such a condition is marked by a suppression of glycolysis and an augmentation of fatty acid oxidation. This aggravates the energy deficiency in cardiomyocytes and might trigger additional harm as a result of the production of excessive reactive oxygen species and inflammatory elements (19). Secondly, proteins are fundamental to the structure and function of cardiomyocytes; insufficient intake can result in impaired regeneration of cardiomyocytes and prolong the time required for myocardial repair (20). Moreover, malnutrition can lead to decreased immune function, increasing the risk of complications such as infections (21), which not only extend hospital stays but can also further worsen patient outcomes.

Systemic nutritional status is closely related to inflammatory responses. Malnutrition can trigger or exacerbate inflammation, characterized by elevated levels of pro-inflammatory cytokines (22). Malnutrition can lead to abnormal immune cell function, such as impaired macrophage and T lymphocyte function, making it difficult for patients to effectively clear cellular debris and inflammatory mediators from the myocardial infarction area, thereby prolonging the duration of the inflammatory response (10). Inflammatory responses induce oxidative stress, creating a vicious cycle that further deteriorates patient outcomes (23). Excessive inflammation may result in cardiac reperfusion damage, myocardial fibrosis, as well as scar formation, affecting the recovery and reconstruction of myocardial function and impacting patient outcomes (24, 25). Regulating inflammatory responses may help reduce myocardial damage after infarction and improve patient outcomes (26). The results of mediation analysis imply that lymphocytes substantially intermediate the connection between GNRI and death from all causes in AMI patients, affecting results both over a brief period (30 days) and an extended period (365 days). While GNRI has a direct effect on mortality, a portion of this effect is mediated through its influence on lymphocyte levels. This indicates that levels of nutrition, exhibited by GNRI, may influence patient outcomes by altering inflammatory processes, which are acknowledged as crucial in the advancement and evolution of AMI. The findings of present study emphasize the possible significance of taking into account inflammatory indicators like lymphocytes while evaluating the prognostic significance of nutritional status in patients afflicted with AMI. The GNRI is a straightforward computation utilizing body weight and blood albumin levels, initially intended to evaluate the state of nutrition of older individuals (27). It not only effectively identifies nutritional risk in elderly patients but is also closely associated with the prognosis of other chronic diseases (28). This study’s results underscore the clinical importance of GNRI in individuals with AMI. The subgroup examination regarding 30-day and 365-day mortality confirms the robustness and consistency of the association. These findings reinforce the potential utility of GNRI in clinical practice for long-term risk stratification and patient management following AMI.

To perform a more comprehensive evaluation of the influence of GNRI, we executed a landmark analysis 20 days after the time of admission. This analysis allowed us to separately examine the influence of GNRI on mortality within the first 20 days after admission and between 20- and 30-days. The landmark analysis underscores the significance of considering the GNRI as a prognostic factor during the early recovery phase of AMI. The significant association between GNRI and mortality within the first 20 days after admission, as well as the trend observed between 20 and 30 days. The landmark analysis underscores the importance of considering GNRI as a prognostic factor for AMI patients throughout their extended recovery phase. The consistent association between GNRI and mortality within the first 240 days after admission, as well as the observed trend between 240 and 365 days, highlights the potential in evaluating long-term prognosis. These results strengthen the necessity for continuous nutritional evaluation and treatment in AMI patients to enhance their recuperation and diminish the mortality probability. Landmark analysis is a commonly used statistical method in survival analysis that allows researchers to reclassify patients at specific time points during follow-up, thereby evaluating the impact of a variable on survival outcomes across different time periods. The benefit of utilizing landmark analysis in this study is its ability to dynamically illustrate the impact of GNRI at different recovery stages, hence enhancing comprehension of nutritional status’s significance throughout the disease trajectory.

In order to optimize the quality of care for AMI patients, clinicians should calculate GNRI at the early stage of the patient’s admission. Once the GNRI score of the patient is determined, they can be classified into high, medium, low, or no nutritional risk groups accordingly, thereby helping medical staff quickly identify those individuals who may require early intervention. For AMI patients determined to have nutritional risk, it is recommended to immediately base on nutritional support, including involving professional dietitians in the treatment plan and formulating highly targeted diet adjustment plans to provide sufficient energy and protein support and promote the repair and regeneration of damaged myocardial tissue. In a double-blind, randomized, placebo-controlled trial targeting patients hospitalized for cardiovascular and pulmonary diseases and with malnutrition, providing oral nutritional supplements effectively improved the patient’s grip strength and was associated with improvements in nutritional and activities of daily living status, providing an important reference for clinical treatment (29). At the same time, considering the impact of the inflammatory state on prognosis, changes in lymphocyte counts and other related biomarkers should be closely monitored to assess the degree of inflammatory response.

This research possesses multiple limitations. First, as a retrospective study based on the MIMIC-IV database, we cannot entirely eliminate the impact of residual confounding variables, such as lifestyle information like smoking and drinking. Despite adjusting for many recognized confounders, there may still exist unmeasured variables that influence the study outcomes. Second, while GNRI is a simple tool for nutritional assessment, it still may not fully reflect the patient’s overall nutritional status. Future prospective studies may employ supplementary nutritional assessment techniques to deliver a more thorough examination of the nutritional condition of AMI patients. The present research exclusively examined the correlation between GNRI and mortality, neglecting other significant health outcomes related to readmission rates along with standard of living. While the mediation analysis indicates that lymphocytes play a role in the relationship between GNRI and mortality, further experimental research is still needed to elucidate the underlying biological mechanisms. Meanwhile, long-term follow-up studies are required to understand how changes in nutritional status over time influence recovery trajectories in AMI patients. Cohort studies tracking GNRI and other nutritional markers alongside clinical outcomes could identify critical periods when nutritional interventions have the greatest impact. This information could inform the timing and intensity of interventions.

In summary, through systematic data analysis and innovative methodological design, this study has revealed a close relationship between GNRI and the prognosis of AMI, highlighting the critical role of nutritional status in AMI. These findings not only provide clinicians with a new biomarker but also offer theoretical support for optimizing nutritional intervention strategies for AMI patients. Subsequent study should corroborate these findings and investigate the practical implications of supplementary dietary therapies to enhance medical outcomes for AMI.

## Data Availability

The datasets presented in this study can be found in online repositories. The names of the repository/repositories and accession number(s) can be found in the article/Supplementary material.

## Footnotes

^1^ https://physionet.org/content/mimiciv/3.0/

^2^ https://www.statsape.com/
